# Hemostatic Tampon to Reduce Bleeding following Tooth Extraction

**Published:** 2012-06-30

**Authors:** M H Kalantar Motamedi, F Navi, E Shams Koushki, R Rouhipour, S M Jafari

**Affiliations:** 1 Trauma Research Center, Baqiyatallah University of Medical Sciences, Tehran, Iran; 2 Scientific Faculty, Azad University of Medical Sciences, Tehran, Iran; 3 General Practitioner, Tehran University of Medical Sciences, Tehran, Iran

**Keywords:** Hemostatic Tampon, Bleeding, Tooth extraction

Dear Editor,

Bleeding following tooth extraction is of concern to patients. Bleeding due to tooth extraction causes discomfort for patients. Proper control of bleeding is thus important. Postextraction oozing is common. However, bleeding usually stops after gauze is placed over the socket and compressed by biting down on the gauze for 30-60 min. In some patients oozing persists. A combination of local antifibrinolytic therapy and a local hemostatic agent has been shown to be effective in preventing postoperative bleeding after oral surgery.

**Fig. 1:**
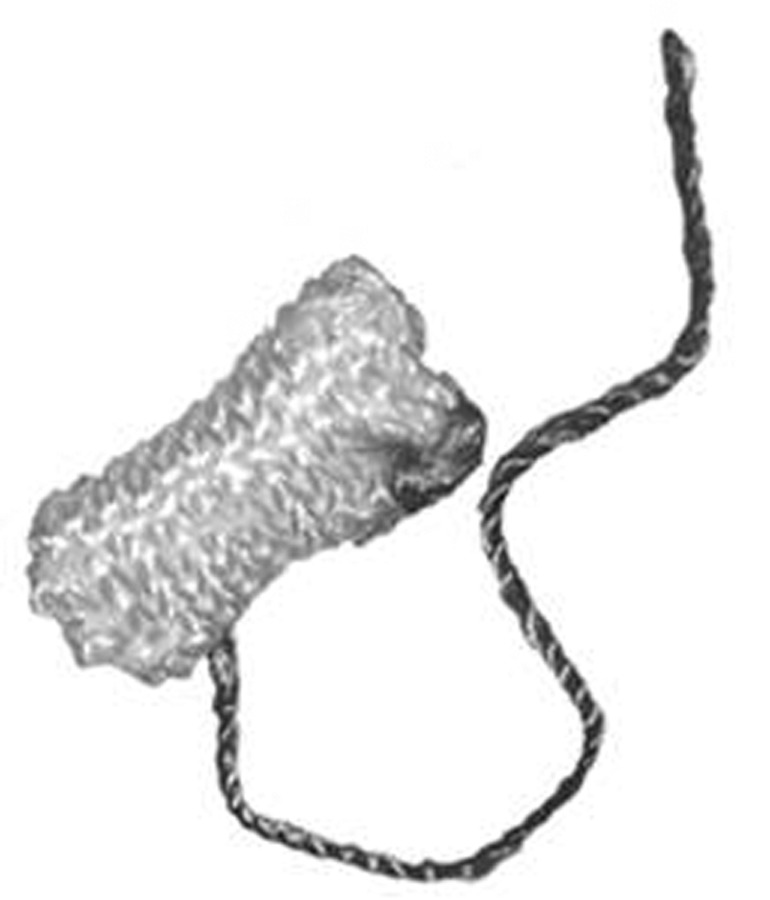
Dental Cell tampon

We assessed the effect of a hemostatic tampon in 50 patients (34 men and 16 women) after tooth extraction. A split-mouth study was done on healthy patients (without congenital bleeding disorders, systemic disease, drug use etc) chosen randomly from patients referring for extraction. Patients requiring at least two symmetrical teeth in one jaw were chosen. Ethics committee approval was obtained, and all pa-tients gave written informed consent. Data included the duration, tooth (molar or premolar), complications, as well as the patient’s gender and age. On one side, a hemostatic tampon was used after extraction (the case group) and on the contralateral side dental gauze impregnated with normal saline was used. The type and amount of anesthetic (3% mepivacain HCl) was identical. Extractions were carried out atraumatically. Simple forceps removal was used; after extractions, the sockets were covered with either normal saline soaked gauze or hemostatic cellulose tampon (Dental Cell, Chitotech, Iran; Figure 1). Pressure was then applied by having the patient bite down on the sponge for 2 minutes. All of extraction sites evaluated for bleeding at 2 and 5 minutes after extractions and then the dressing was removed. If bleeding persisted, the gauze was retained and the patient was sent home and contacted the next morning. The duration of active bleeding was assessed both after the first tooth extraction and after the second tooth extraction on the contralateral side (the next week). Both teeth were extracted by one practitioner. The acquired variables (duration of active bleeding, continued bleeding, cessation of bleeding, gender and age) were analyzed (SPSS software, Version 11, USA) and compared by T-test, the Exact Fisher and Chi-Square tests (Values of p=0.05 were considered significant).

**Fig. 2:**
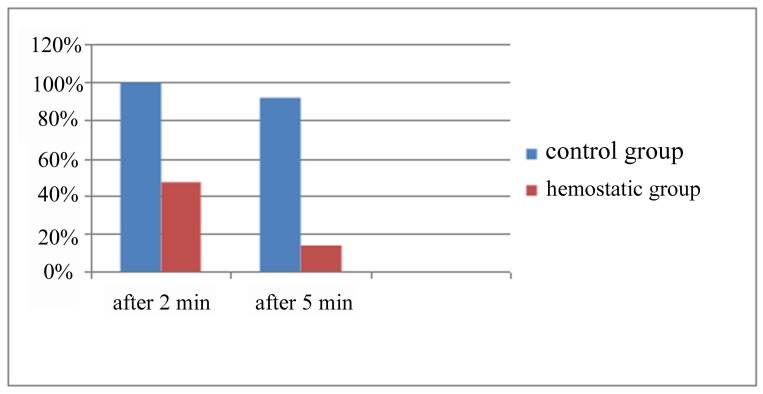
Percentage of patients with bleeding using regular gauze and cellulose gauze.

In this study, 34/50 patients (68%) were male and 16/50 patients (32%) were female. The mean age was 35.8±9.75 years, ranging from 20-57 years (no significance). Within 2-5 minutes after tooth removal, 92% of subjects continued bleeding on the side that gauze was impregnated with normal saline; while on the side which the hemostatic tampon was used, only 14% continued to bleed. Cessation of bleeding in sockets using gauze impregnated with normal saline, stopped after 10.04±2.95 minutes, while after using the hemostatic tampon, this time period decreased to 2.73±0.9 minutes. The difference between the two groups was significant. After 2 minutes on the control side, 100% of the patients had bleeding and in 48% on the hemostatic tampon side had continued bleeding; and difference between the two groups (continued bleeding) was also significant. Attributable risk for bleeding after 2 minutes was 52%. After 5 minutes, 92% of patients continued to bleed on the control side and in 14% of patients on the hemostatic tampon side. The difference between the two groups was significant (Relative Risk: 6.5 and attributable risk was 78%). The difference (bleeding after 2 minutes and after 5 minutes) between the two groups was significant (Figure 2).

There are several ways to reduce postextraction bleeding. Gaspar used a combination of local antifibrinolytic therapy and a local hemostatic agent to prevent postoperative bleeding after oral surgery in patients treated with anticoagulants.[1] The HemCon Dental Dressing (HDD) hemostatic oral wound dressing derived from the US military has been proven to be a clinically effective hemostatic that significantly shortens bleeding time following oral surgery procedures for all patients.[2] Several studies showed the use of fibrin glue was a safe and cost-effective tool to treat patients with severe bleeding disorders. Fibrin adhesive is as effective as the resorbable oxycellulose dressing in preventing post-extraction hemorrhage.[2][3][4] Dental cell is a local hemostatic action depends on the binding of hemoglobin to oxycellulose, allowing the dressing to expand into a gelatinous mass, which acts both as scaffolding for clot formation and as a clot stabilizer. The cellulose tampon is a soft dressing that is made of cellulosic fabric. It does not stick to the clot so it may easily be removed after hemostasis. In our study, after 2 and 5-minutes postextraction, the hemostatic tampon significantly reduced bleeding time. According to the results of this study, use of a hemostatic tampon significantly decreased the hemostasis time when compared with the normal saline soaked dressings thus reducing discomfort for patients. Use of a hemostatic tampon after tooth extraction did not require gauze to be placed after removal of the tampon. This may be an alternative to gauze in patients that cannot tolerate retaining gauze in their mouth for a longer time period.
